# A novel *Kluyveromyces marxianus* strain with an inducible flocculation phenotype

**DOI:** 10.1186/2191-0855-2-38

**Published:** 2012-07-29

**Authors:** Juan A Vallejo, Manuel Serrat, Irasema Pérez-Portuondo, Angeles Sánchez-Pérez, Jose M Ageitos, Tomas G Villa

**Affiliations:** 1 Department of Microbiology and Parasitology, Faculty of Pharmacy, University of Santiago de Compostela, Campus Sur 15782, 15706, Santiago de Compostela, Spain; 2 Industrial Biotechnology Studies Center (CEBI), University of Oriente, Ave. Patricio Lumumba s/n, 90500, Santiago de Cuba, Cuba; 3 Discipline of Physiology and Bosch Institute, School of Medical Sciences, University of Sydney, Sydney, NSW, 2006, Australia

**Keywords:** *Kluyveromyces marxianus*, Inducible flocculent phenotype, Polygalacturonase, Ethanol

## Abstract

Flocculation is a very useful phenotype for industrial yeast strains, since it facilitates cell harvest and represents an easy way of cell immobilization in continuous fermentation processes. The present work represents the first time that an inducible flocculation phenotype has been generated in a non flocculent strain of *Kluyveromyces marxianus*. This was accomplished by expressing *Saccharomyces cerevisiae* FLO5 gene in *K. marxianus* CECT 11769 strain. The FLO 5 gene was placed under the control of an EPG promoter, not repressed by glucose and induced by anoxia. Our experimental approach successfully generated two novel *K. marxianus* flocculent phenotypes: one inducible and one constitutive. The constitutive phenotype originated from deletions in the FLO5 promoter region, indicating the existence of putative upstream repressor site involved in oxygen regulation of the EPG1 promoter. The novel strains here generated had a unique set of characteristics that provided an advantage, over the wild-type strain, for the industrial co-production of ethanol and polygalacturonase.

## Introduction

Yeast flocculation is defined as an asexual and reversible Ca^2+^-dependent cellular aggregation that generates fast-sedimenting flocs containing high numbers of yeast cells (Bony et al. [1997]; Stratford, [1989]). Under adverse conditions, the innermost cell layer lyses thus providing nutrients for the cells in the outer layer (Hercker et al. [2004]). Yeast growth under flocculating conditions, while often slow due to the difficulty in the uptake of nutrients, protects the cells from toxic substances accumulating in the media (Van Mulders et al. [2009]). Thus, the floc phenotype confers resistance to ethanol and oxidative stresses (Smukalla et al. [2008]).

Flocculation is a very useful property for an industrial yeast strain, since it allows the separation of the biomass from the culture broth in a fast, cheap and innocuous way. Additional advantages include: i) flocculent yeast can be used in high density reactors with high productivity and reduced fermentation times (Teixeira et al. [1990]); ii) they can be used under different fermenter configurations with suspended biomass, thus avoiding the risk of biomass washout (Domingues et al. [2000b]); and iii) flocculent yeast cultures have lower contamination rates, due to the culture’s high metabolic activity (Domingues et al. [2000a]), when the yeast are grown in continuous fermentations conditions.

Yeast flocculation may be ascribed to two main categories: i) sugar-sensitive adhesion (lectin-like) and ii) sugar-insensitive adhesions. The latter mediated by adhesins that bind to peptides instead of sugars so increasing the cellular surface hydrofobicity, and promoting either cell/cell or cell/surface interactions (Kang and Choi, [2005]). The sugar-sensitive adhesions are the subject of the present work and follow the rules proposed by Miki et al. ([1982]). The lectin-like molecules present on the yeast cell surface, and activated by Ca^2+^ (Miki et al. [1982]; Stratford [1989]), interact with the α-mannan carbohydrates of adjoining cells, thus causing the formation of three-dimensional nets.

The yeast flocculation genes described so far are the FLO genes, encoding proteins also known as flocculins. FLO1 is the best known; it codes for the protein Flo1p, and has two alleles, FLO2 and FLO4. Genes FLO5, FLO9 and FLO10 encode proteins with 96, 94 and 58% homology to Flo1p, respectively. The six genes, either alone or in combination, are responsible for the Flo1 phenotype. On the other hand, the *Lg-FLO1* gene is a homologue of FLO1 and is responsible for the NewFlo phenotype. Finally, the protein encoded by the FLO11 gene only displays 37% homology to the Flo1p protein and is responsible for the above mentioned sugar-insensitive adhesion phenotype. FLO11 gene expression has also been associated with properties such as agar invasion, pseudohyphae formation and substrate adhesion (Goossens and Wilaert, [2010]).

*K. marxianus* is yeast species with high biotechnological potential, due to its ability to use a variety of substrates and its high growth rate under aerobic conditions (Fonseca et al. [2008]). The aim of the present work was to engineer an inducible flocculation phenotype into the non flocculent CECT 11769 strain of *K. marxianus* (Serrat et al. [2002]) for the purpose of industrial co-production of endopolygalacturonase (EPG) and ethanol (Serrat et al. [2004]). We chose the CECT 11769 strain because, while displaying the typical properties of the species, its EPG production is not repressed by high glucose concentrations; additionally, this strain exhibits one of the highest specific growth ratios so far described (Serrat et al. [2011]). The flocculating phenotype is an important trait in ethanol-producing yeast strains, since it protects the yeast from the negative effects of ethanol (Hu et al. [2005]) as well as providing a cheap form of yeast cell immobilization for continuous fermentations, with more volumetric productivity. In this way we could overcome the lower ethanol tolerance of *K. marxianus*, as compared to *S. cerevisiae*, and its lower EPG production, as compared to filamentous fungi (Fonseca et al. [2008]). Furthermore, the currently used procedure of yeast cell immobilization, using agglutinating substances, for ethanol production, is often detrimental, rather than advantageous. This is because this procedure can affect the ethanol production itself (or another primary metabolite), apart from directly adding an extra cost to the industrial procedure, where commercial margins are already narrow (Zhao et al. [2009]). Here we report the construction of a *K. marxianus* CECT 11769 strain with an inducible flocculating phenotype. This was accomplished by expressing the *S. cerevisiae* FLO5 gene under the control of *K. marxianus* CECT 11769 native EPG1 promoter. This promoter allows the induction of the flocculation phenotype by anaerobic conditions, without glucose repression. FLO1 and FLO9 genes were not considered because, when over-expressed, they reduced the yeast growth ratio (Van Mulders et al. [2009]).

## Materials and methods

### Microbial strains, plasmids and culture conditions

The microbial strains used in this work were *Escherichia coli* TOP10 (Invitrogen, Carlsbad, CA, USA), *S. cerevisiae* 99R (Yeast Genetic Stock Center) and *K. marxianus* CECT 11769 (Serrat et al. [2002]).

*E. coli* TOP10 cells were cultured in LB medium (10 g/L tryptone, 5 g/L yeast extract and 10 g/L NaCl) supplemented with Zeocin (Invitrogen) at a concentration of 25 μg/mL. *S. cerevisiae* 99R and *K. marxianus* CCEBI were grown in YPD medium (10 g/L yeast extract, 10 g/L peptone and 20 g/L glucose) supplemented with Zeocin (400 μg/mL) to select recombinant *K. marxianus* cells. YNB-glucose (6.7 g/L Yeast Nitrogen Base without amino acids plus 20 g/L glucose) and YPD10 medium (10 g/L yeast extract, 10 g/L peptone and 100 g/L glucose) were used in *K. marxianus* flocculation test and fermentations.

### Construction of FLO5 expression vector (pEPG-FLO5)

**Figure 1:**
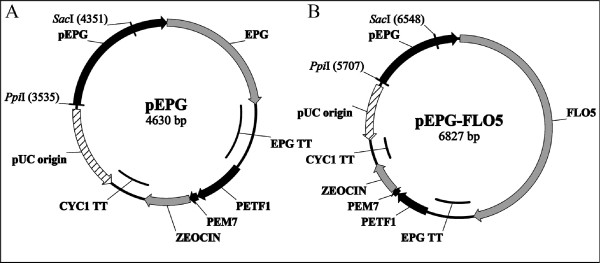
**Construction of the*K. marxianus*FLO5 expression vector (pEPG-FLO5)**. **A**) The intermediate plasmid pEPG was designed by introducing an EPG1 cassette into the pGAPZα vector. **B**) The pEPG-FLO5 expression vector was constructed by replacing the EPG gene, in pEPG, with the FLO5 gene. The plasmid map denotes the position of the recognition sites for the restriction enzymes *Sac*I, used for plasmid linearization to achieve homologous recombination, and *Ppi*I, the enzyme used to linearize the DNA for random genomic insertion.

Construction of the FLO5 expression vector was carried out in two steps. The first step involved engineering a *K. marxianus* expression vector containing the EPG1 cassette (pEPG, Figure 1A). For this purpose, the EPG1 cassette was PCR-amplified from *K. marxianus* CECT 11769 genomic DNA, using High-Fidelity DNA Polymerase (Bio-Rad, Hercules, CA, USA) and a primer pair containing recognition sites for the restriction enzyme *Bcl*I (Forward: 5' ctgatcagGAGGCCTGTCCGATTATTAAAC 3'; Reverse: 5' ctgatcagCTGCAGAGACATGTATCATTTTC 3'). The amplified DNA fragment was then digested with *Bcl*I (New England Biolabs, Ipswich, MA, USA) and ligated to *Bgl*II/*BamH*I digested, dephosphorylated (using Shrimp Alkaline Phosphatase, Takara, Japan) pGAPZα vector (Invitrogen). The pEPG expression plasmid thus obtained was used to transform *E. coli* TOP10 competent cells, and the correct DNA orientation selected by digestion with the restriction enzyme *Sma*I (Takara). The second step entailed the construction of the pEPG-FLO5 expression vector (Figure 1 B). This was achieved by replacing the EPG gene with the FLO5 gene. The FLO5 gene was PCR-amplified from the *S. cerevisiae* 99R genome, using High-Fidelity DNA Polymerase (Bio-Rad) and specific primers. The forward primer included part of the Kozak consensus sequence (5' aaaaaaaATGACAATTGCACACCACTGC 3') while the reverse primer included a recognition site for the restriction enzyme *Asci*I (5' ggcgcgccATGACAATTGCACACCACTGC 3'). The PCR-amplified DNA fragment, corresponding to FLO5, was digested with *Asci*I and ligated to *Asci*I digested PCR-amplified pEPG fragment. PCR-amplification of pEPG was carried out with a forward primer containing the recognition site for the restriction enzyme *Asci*I (5' ggcgcgccTAATAGCGGAGCCTTCTGTTAA 3’) and a reverse primer containing part of the sequence for the Kozak consensus sequence (5' tttttttGGTTTCTGAGCTTAC 3').

The *K. marxianus* FLO5 promoter was PCR-amplified using High-Fidelity DNA Polymerase (Bio-Rad) and specific primers (Forward 5' TGTCCGATTATTAAACTTGC 3' and reverse 5' GAAATGTGCCTGATGAACT 3'). The DNA fragment thus amplified included the FLO5 promoter and the start of the FLO5 gene.

All plasmids constructed in this study were subjected to DNA sequencing before use, and DNA sequence alignment was carried out with Vector NTI (Invitrogen).

### Transformation of *K. marxianus*

The pEPG-FLO5 plasmid was linearized, using either the restriction enzymes *Sac*I (Takara), to induce homologous recombination or *PpiI* (Fermentas, Glen Burnie, Ma, USA), to induce random DNA insertion, and introduced into chemically-competent *K. marxianus* CECT 11769 cells, as described by Abdel-Banat ([2010a]) and the recombinant colonies obtained were selected by Zeocin resistance. The recombinant colonies were confirmed by PCR-amplification of the recombinant genomic DNA with the EPG1 cassette primers and the FLO5 primers described above.

### Flocculation assays

For flocculation assays, the *K. marxianus* recombinant clones were incubated, either in aerobic or anaerobic conditions, in two different types of media (YPD or YNB plus glucose, respectively) and two different temperatures (30°C and 42°C). Aerobic cultures were grown in 50 mL batches, in 250 mL flasks, with shaking (200 rpm); whereas the anaerobic cultures were grown in 50 mL tubes, containing 45 mL of medium, with gentle shaking (50 rpm). The media were inoculated with 2% (v/v) of cell suspension in water, from a fresh culture in YPD broth, and incubated either at 30°C or 42°C until the beginning of stationary phase. Flocculating cells were visualized in a 50 mL graduate cylinder by measuring the amount of sedimenting cells present in the suspension after vigorous vortexing.

### Yeast growth assays

One colony from each flocculent phenotype, as well as from the wild-type, was selected and inoculated into a 50 mL flask, containing 10 mL YPD medium, and incubated for 16 hours at 30°C in a rotary shaker at 250 rpm. One milliliter of the above cultures was then inoculated into a 250 mL flask, containing 50 mL of YPD medium, and incubated for 5 hours under the conditions described above. The cells were collected by centrifugation and successively washed with 100 mM EDTA, 30 mM EDTA and distilled water (all under sterile conditions). The O.D., at 600 nm, of the cultures was then measured and equal cell amounts inoculated into three 250 mL flasks containing 50 mL YPD medium. The biomass thus generated was determined and estimated as dry cell weight produced after 24 h of growth at either 30°C or 42°C.

### *K. marxianus* fermentations

One colony from each yeast strain was inoculated into 50 mL flasks containing 10 mL of YPD medium and incubated at 30°C for 16 h with shaking (250 rpm). One milliliter from each of the cultures was inoculated into a 250 mL flask, containing 50 mL of YPD medium, and incubated for 5 hours under the above conditions. The cells were then collected by centrifugation and successively washed with 100 mM EDTA, 30 mM EDTA and distilled water (with all procedures carried out under sterile conditions). Finally, the cells were re-suspended in 5 mL of distilled water and their O.D. at 600 nm determined. The cellular suspension was then adjusted to OD_620_ = 100 and 50 mL Falcon tubes, containing 45 mL of YPD10, were inoculated with enough yeast cells to obtain an initial OD_600_ = 1. The cultures were statically grown, at either 30 or 42°C, for 12 and 24 hours. These fermentation assays were carried out in triplicate. At the end of their incubation time, the cells were collected by centrifugation and the growth supernatants stored at -20°C until use.

### Ethanol production and fermentation efficiency

The ethanol concentration of the above supernatants was measured using an Ethanol Determination Kit (R-Biopharm, Darmstadt, Germany) following the manufacturer's instructions. Reducing sugars in the medium were determined according to the Somogyi-Nelson (Nelson, [1944]) procedure. The fermentation efficiency was estimated as the maximal theoretical ethanol yield per substrate consumed.

### EPG assay

EPG activity was qualitatively detected on plates containing 6.7 g/L of YNB, 5 g/L of glucose, 5 g/L of polygalacturonic acid (Sigma), and 20 g/L of bacteriological agar (Difco), with the pH adjusted to 5.4. Plates were incubated at 30°C for 72 h, and the enzyme activity visualized by the diameter of the hydrolysis haloes produced after the plates had been flooded with 6 M HCl (Zink and Chatterjee, [1985]). The EPG activity was measured in the culture supernatant by estimating the increase in the reducing power, using 1% (w/v) polygalacturonic acid in 50 mM sodium acetate buffer (pH 5.0) as the substrate, at 37°C for 10 min (Nelson, [1944]). A typical reaction mixture contained 400 μL of substrate and 100 μL of the appropriate supernatant dilution. One unit of EPG activity was defined as the amount of enzyme needed to produce 1 μmol/min of galacturonic acid, or equivalent reducing power, under these conditions.

## Results

### Engineering *K. marxianus* strains that display a flocculation phenotype

**Figure 2:**
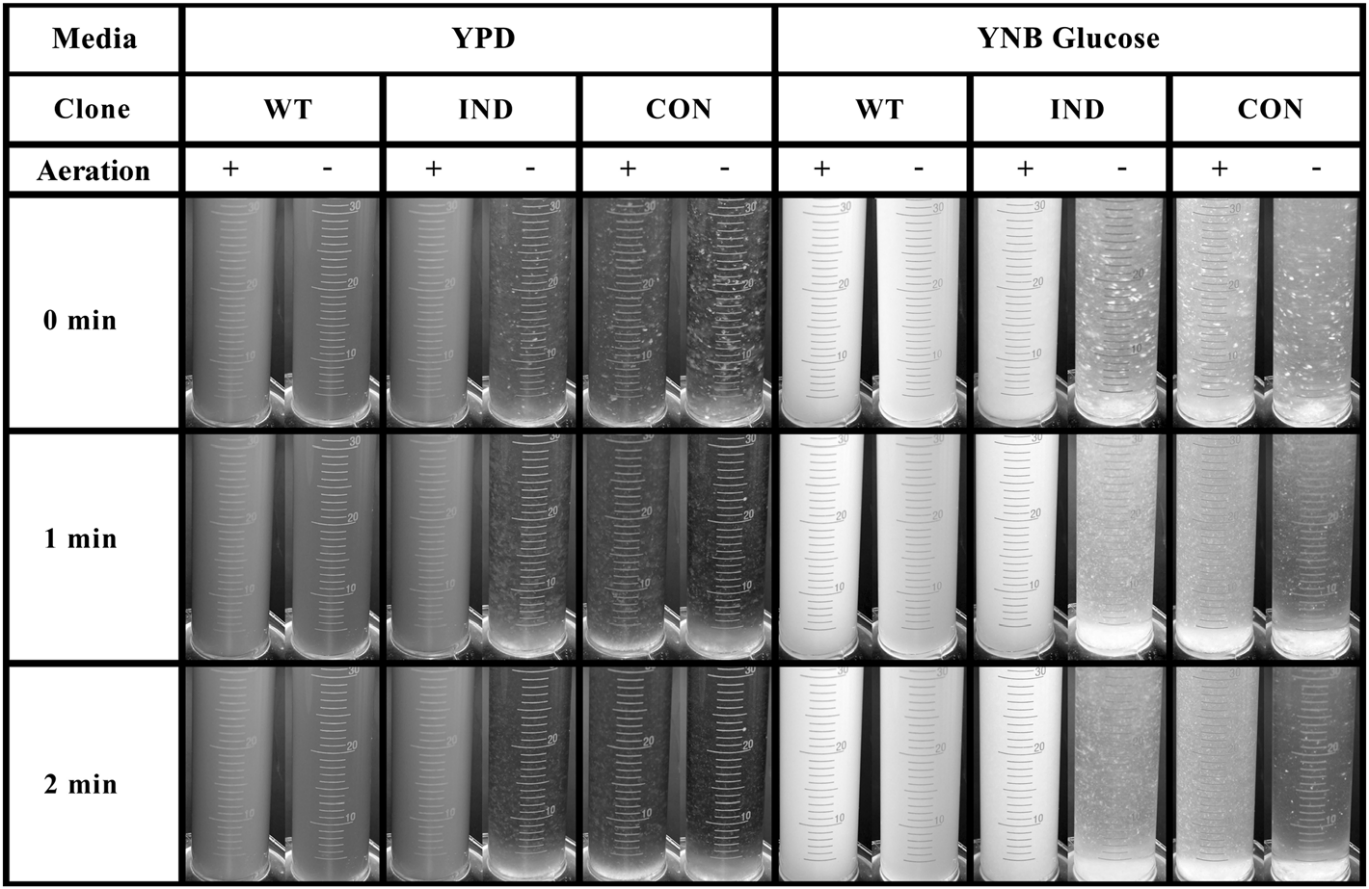
**Flocculation assays of*K. marxianus*strains.** The WT (Wild type); IND (inducible phenotype), and CON (constitutive phenotype) yeast were grown in either aerobic (aeration +) or anaerobic (aeration -) conditions and subjected to flocculation, as described in Materials and Methods. The yeast cultures were then observed at 0, 1 or 2 minutes after vortexing.

**Figure 3:**
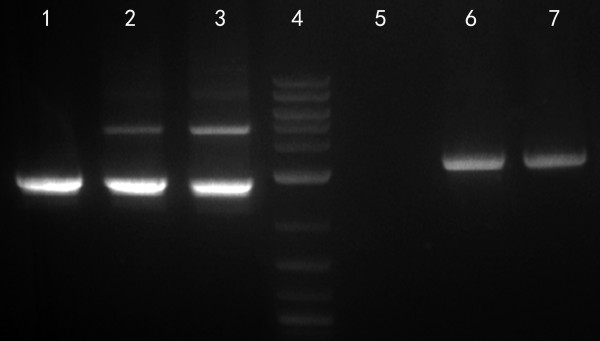
**PCR-amplification confirms the presence of both the FLO5 and EPG1 cassettes in the recombinant yeast strains.** Lane 1: Wild type strain containing the EPG1 cassette (2772 bp). Lane 2: Inducible flocculent phenotype containing the FLO5 (4921 bp) and EPG1 cassettes. Lane 3: Constitutive flocculent phenotype displaying the FLO5 and EPG1 cassettes. Lane 4: Molecular Weight Marker (2-Log DNA Ladder, New England Biolabs). Lane 5: The wild type strain does not contain a FLO5 gene. Lane 6: The FLO5 gene was PCR-amplified (3227 bp) from the inducible flocculent phenotype Lane 7: The FLO5 gene amplified from the constitutive flocculent phenotype.

**Figure 4:**
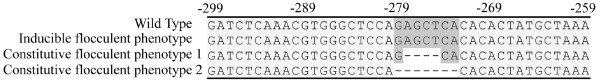
**Alignment of the relevant region of the sequenced EPG1 promoter, driving the expression of FLO5 in the recombinant yeast, with the original EPG1 promoter found in the wild type*K. marxianus*strain.** The nucleotide positions upstream of the ATG are presented as negative numbers and the differences between the promoter sequences are shown in grey.

The aim of the present work was to engineer an inducible flocculation phenotype into the CECT 11769 strain of *K. marxianus.* For this purpose, we constructed an integrative expression vector (pEPG-FLO5; Figure 1B) containing the FLO5 gene from *S. cerevisiae* under the control of EPG1, a promoter not repressed by glucose and induced by anoxic conditions. The expression plasmid was built in two steps. The first step encompassed the construction of plasmid pEPG, containing the EPG1 cassette (Figure 1A), and this was followed by substitution of the EPG gene by the FLO5 gene. The resulting expression vector, pEPG-FLO5, was linearized with either *Ppi*I (a restriction enzyme with a recognition site upstream of the EPG1 promoter (Figure 1B), or with *Sac*I (this enzyme has a recognition site in the middle of the EPG1 promoter; Figure 1B). Transformation of *K. marxianus* with the *Ppi*I-linearized plasmid successfully generated a novel CECT 11769 strain with an inducible flocculent phenotype (Figure 2). On the other hand, transformation of *K marxianus* with the *Sac*I-linearized expression plasmid resulted in the creation of a novel CECT 11769 strain with a constitutively-expressed flocculent phenotype (Figure 2). Both phenotypes were flocculent when grown at either 30°C or 42°C and, in both cases, flocculation was abrogated when Ca^2+^ was absent from the culture media (data not shown). There is, however, an outstanding difference regarding the transformation efficiency of the two linearized plasmids. Whereas the *Ppi*I-linearized plasmid (inserted randomly into the host DNA) produced 347 transformed colonies per microgram of DNA, the transformation efficiency of the *Sac*I-linearized plasmid (inserted by homologous recombination) was only 1.4 transformed colonies per microgram of DNA. We used PCR-amplification (see Materials and Methods) to confirm that all the recombinant colonies exhibiting a flocculent phenotype contained not only the FLO5 cassette integrated in their genome (4.9 kb band; Figure 3), but also the EPG1 cassette (2.8 kb DNA fragment; Figure 3). We also confirmed that all the recombinant flocculent strains harbored the FLO5 gene in their genomes (3.2 kb DNA fragment; Figure 3). We used DNA sequencing to investigate the possibility that the unexpected constitutive flocculent phenotype resulted from either a mutation or a deletion in the pEPG1 promoter. As seen in Figure 4, the promoter driving the expression of FLO5 in the inducible flocculent yeast was identical to the wild-type EPG1 promoter, but this was not the case for the yeast displaying the flocculent constitutive phenotype, in which a deletion had occurred (Figure 4). All of the constitutive strains analyzed were missing a DNA fragment spanning 4 to 7 base pairs in an area (-273 to -279 upstream of the ATG) located within a possible regulation box next to the TATA box.

### Cell growth and production of ethanol and EPG by the recombinant strains

**Figure 5:**
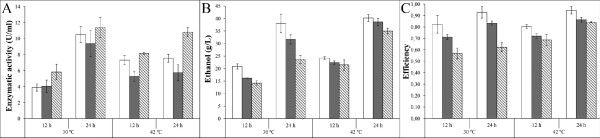
**Endopolygalacturonase (EPG) enzymatic activity (A), ethanol production (B), and overall fermentation efficiency (C) of the three*K marxianus*CECT 11769 strains.** Open bars represent the wild type strain, gray bars the inducible flocculent phenotype, and stripped bars the constitutive flocculent phenotype. Error bars depict Standard deviation.

We analyzed the growth rate and the ethanol and EPG production of both the constitutive and the inducible flocculating phenotypes, as compared to the wild-type. As shown in Table 1, the strain constitutively expressing the flocculent phenotype reached a lower cell density that the inducible strain, with the latter showing comparable growth values to those displayed by the wild-type strain. On the other hand, the constitutive phenotype generated more EPG enzymatic activity (Figure 5A) than the other two strains, independently of the temperature at which the yeast were grown.

**Table 1:** **Growth of the three*K marxianus*CECT 11769 strains at 30 and 42°C**

**Temperature**	**30°C**	**42°C**
Wild Type	7.1 ± 0.14	6.7 ± 0.14
Inducible phenotype	6.8 ± 0.56	6.2 ± 0.28
Constitutive phenotype	4 ± 0.56	4.2 ± 0.28

The growth was measured as the yeast dry weight (g/L).

Additionally, expression of the flocculent phenotype appears to negatively affect ethanol production, with both recombinant strains producing less ethanol than the wild-type when incubated at 30°C; but the difference was minimal when the yeast were grown at 42°C (Figure 5B). Accordingly, the fermentation efficiency (estimated as the maximal theoretical ethanol yield per substrate consumed) followed the same pattern as the ethanol production by the three strains (Figure 5C).

## Discussion

Yeasts strains with the ability to flocculate are very useful in industrial applications, as the flocculent phenotype allows for an easier, and cheaper, way of separating the yeast culture from the final, economically-important, fermentation product (Ratledge and Kristiansen, [2001]); and also because of the higher production rate and cellular densities obtained with these strains (Teixeira et al. [1990]). As a result of this, flocculent strains are ideally suited for use in continuous fermentative processes (Domingues et al. [2000b]). However, the flocculent phenotype negatively affects the yeast growth (Zhao et al. [2009]), therefore an inducible flocculent phenotype would be most desirable to use in fermentation procedures (Govender et al. [2010]).

The present work describes the successful construction of a novel recombinant *K marxianus* CECT 11769 yeast strain with an inducible flocculating phenotype, for the industrial production of EPG and ethanol. This was achieved by engineering a yeast expression vector containing the FLO5 gene, from *S. cerevisiae,* under the control of the EPG1 promoter. This promoter originates from the CECT 11769 strain and has the advantages of not being repressed by glucose (even in high concentrations, such as 100 g/L; Serrat et al. [2002]), as well as being repressed under aerobic conditions. These two properties make this promoter ideally suited for the modulation of yeast flocculent phenotypes in industrial fermentations. In this way, we could use this novel inducible flocculating *K. marxianus* strain in two-steps fermentations. In the first fermentation step, the yeasts are grown, under aerobic conditions, to high cell densities (the aerobic conditions would prevent flocculation, hence favoring cell growth). On the other hand, the second fermentation step involves the production of ethanol and EPG, under anaerobic conditions (producing yeast flocculation at just the right time). This second step could be carried out continuously.

Analyses of the recombinant *K. marxianus* clones obtained revealed that those generated by a processes favoring homologous recombination (when the plasmid DNA was linearized with a restriction enzyme with a cleavage site inside the EPG1 promoter region) resulted in strains displaying the flocculent phenotype in a constitutive manner. On the other hand, the clones generated by a process favoring random recombination (plasmid linearized with a restriction enzyme with a cleavage site located before the start of the promoter) produced strains with inducible flocculent phenotypes. The latter also produced a far higher number of recombinant colonies than the former, in agreement with the results obtained by Nonklang et al. ([2008]) indicating that this yeast species has a strong preference for random over homologous recombination processes. DNA sequencing of the promoter regions from the recombinant yeast revealed that all the clones constitutively expressing FLO5 contained a deletion (4 to 7 bp in length) at position -273 to -279 upstream of the ATG, in a DNA region encompassing a novel putative repressor site (Figure 4), next to the TATA box. This deletion did not appear in the inducible clones, with promoter sequences identical to that of the wild-type strain. This upstream repressor site is closely related to the oxygen repression mechanism that regulates the EPG1 promoter and this could explain why the flocculent phenotype is not repressed under aerobic conditions. Some of these upstream repressor sites involved in oxygen regulation are well characterized in *S. cerevisiae* (Kwast et al. [1998]) and the deletion reported here could be due to a mistake in the impaired homologous recombination mechanisms of this yeast species. Both flocculating phenotypes were stable at 42°C, this is contrary to the data reported by Nonklang et al. ([2009]), indicating that the flocculation phenotype generated by expression of the FLO5 gene is sensitive to temperatures above 40°C.

As shown in Table 1, the constitutive flocculent phenotype displayed slower growth, as indicated by its lower biomass production, than both the inducible and the wild-type strains. This is not surprising, as it had been previously shown by Zhao et al. ([2009]) that expression of the flocculent phenotype interferes with the ability of the yeast to grow. This is the reason why our aim centered on the development of a *K. marxianus* CECT 11769 strain displaying an inducible flocculent phenotype.

EPG is an enzyme produced by *K. marxianus* under stress conditions. This type of enzyme is used by pectinolytic microorganisms to soften plant tissues and get access to the nutrients located inside the plant cells (Blanco et al. [1999]). The constitutive phenotype produced high amounts of EPG, at the two temperatures assayed (Figure 5A), and this could be a result of the higher stress conditions within the floc, as compared to cells growing planktonically.

The inducible flocculating phenotype yielded higher ethanol production that the constitutive strain (Figure 5B and C), but neither of the flocculent phenotypes could match the level of ethanol production by the wild-type when the yeast were grown at 30°C. On the other hand, the differences in yield were greatly reduced when the yeasts were grown at 42°C, with the inducible yeast strain producing a very similar amount of ethanol as the wild-type strain. These results indicate that the inducible strain could be successfully, and advantageously, used in fermentative processes taking place at high temperatures, which are of industrial interest because the use of higher temperatures results in the reduction of both the cost and the risk of microbial contamination (Abdel-Banat et al. [2010b]).

In conclusion, the present work resulted in the generation of two novel *K marxianus* CECT 11769 flocculent phenotypes, one constitutive and the other inducible, that successfully produce ethanol and EPG. Apart from the advantage that flocculation represents in industrial fermentations, the constitutive phenotype produces more EPG enzymatic activity than the wild-type strain, whereas the inducible phenotype produces similar amounts of ethanol as the wild-type.

## Competing interests

The authors declare that they have no competing interests.

## References

[B1] Abdel-Banat BMA Nonklang S Hoshida H Akada R Random and targeted gene integrations through the control of non-homologous end joining in the yeast Kluyveromyces marxianus Yeast 2010 27 29 39 19894210 10.1002/yea.1729

[B2] Abdel-Banat BMA Hoshida H Ano A Nonklang S Akada R High temperature fermentation: how can processes for ethanol production at high temperatures become superior to the traditional process using mesophilic yeast? Appl Microbiol Biotechnol 2010 85 861 867 10.1007/s00253-009-2248-5 19820925

[B3] Blanco P Sieiro C Villa TG Production of pectic enzymes in yeasts FEMS Microbiol Lett 1999 175 1 9 10.1111/j.1574-6968.1999.tb13595.x 10361703

[B4] Bony M Thines-Sempoux D Barre P Blondin B Localization and cell surface anchoring of the Saccharomyces cerevisiae flocculation protein Flo1p J Bacteriol 1997 179 4929 4936 9244284 10.1128/jb.179.15.4929-4936.1997 PMC179343

[B5] Domingues L Lima N Teixeira JA Contamination of a High-cell-density continuous Bioreactor Biotechnol Bioeng 2000 68 584 587 10.1002/(SICI)1097-0290(20000605)68:5<584::AID-BIT14>3.0.CO;2-1 10797246

[B6] Domingues L Vicente AA Lima N Teixeira JA Applications of yeast flocculation in biotechnological processes Biotechnol Bioprocess Eng 2000 5 288 305 10.1007/BF02942185

[B7] Fonseca GG Heinzle E Wittmann C Gombert AK The yeast Kluyveromyces marxianus and its biotechnological potential Appl Microbiol Biotechnol 2008 79 339 354 10.1007/s00253-008-1458-6 18427804

[B8] Goossens K Willaert R Flocculation protein structure and cell-cell adhesion mechanism in Saccharomyces cerevisiae Biotechnol Lett 2010 32 1571 1585 10.1007/s10529-010-0352-3 20640875

[B9] Govender P Bester M Bauer FF FLO gene-dependent phenotypes in industrial wine yeast strains Appl Microbiol Biotechnol 2010 86 931 945 10.1007/s00253-009-2381-1 20013339

[B10] Herker E Jungwirth H Lehmann KA Maldener C Froehlich K Wissing S Buettner S Fehr M Sigrist S Madeo F Chronological aging leads to apoptosis in yeast J Cell Biol 2004 164 501 507 10.1083/jcb.200310014 14970189 PMC2171996

[B11] Hu C Bai F An L Effect of flocculence of a self-flocculating yeast on its tolerance to ethanol and the mechanism Shengwu Gongcheng Xuebao 2005 21 123 128 15859341

[B12] Kang S Choi H Effect of surface hydrophobicity on the adhesion of S. cerevisiae onto modified surfaces by poly(styrene-ran-sulfonic acid) random copolymers Colloids Surf B 2005 46 70 77 10.1016/j.colsurfb.2005.08.017 16256322

[B13] Kwast KE Burke PV Poyton RO Oxygen sensing and the transcriptional regulation of oxygen-responsive genes in yeast J Exp Biol 1998 201 1177 1195 9510529 10.1242/jeb.201.8.1177

[B14] Miki BLA Poon NH James AP Seligy VL Possible mechanism for flocculation interactions governed by gene FLO1 in Saccharomyces cerevisiae J Bacteriol 1982 150 878 889 7040343 10.1128/jb.150.2.878-889.1982 PMC216441

[B15] Nelson N A photometric adaptation of the Somogyi method for the determination of glucose J Biol Chem 1944 153 375 380

[B16] Nonklang S Abdel-Banat BMA Cha-aim K Moonjai N Hoshida H Limtong S Yamada M Akada R High-temperature ethanol fermentation and transformation with linear DNA in the thermotolerant yeast Kluyveromyces marxianus DMKU3-1042 Appl Environ Microbiol 2008 74 7514 7521 10.1128/AEM.01854-08 18931291 PMC2607150

[B17] Nonklang S Ano A Abdel-Banat BMA Saito Y Hoshida H Akada R Construction of flocculent Kluyveromyces marxianus strains suitable for high-temperature ethanol fermentation Biosci Biotechnol Biochem 2009 73 1090 1095 10.1271/bbb.80853 19420680

[B18] Ratledge C Kristiansen B Basic Biotechnology 2001 Second Edition, 584

[B19] Serrat M Bermudez RC Villa TG Polygalacturonase and ethanol production in Kluyveromyces marxianus. Potential use of polygalacturonase in foodstuffs Appl Biochem Biotechnol 2004 117 49 64 10.1385/ABAB:117:1:49 15126703

[B20] Serrat M Bermudez RC Villa TG Production, purification, and characterization of a polygalacturonase from a new strain of Kluyveromyces marxianus isolated from coffee wet-processing wastewater Appl Biochem Biotechnol 2002 97 193 208 10.1385/ABAB:97:3:193 11998843

[B21] Serrat M Rodriguez O Camacho M Vallejo JA Ageitos JM Villa TG Influence of nutritional and environmental factors on ethanol and endopolygalacturonase co-production by Kluyveromyces marxianus CECT 11769 Int Microbiol 2011 14 41 49 22015701 10.2436/20.1501.01.134

[B22] Smukalla S Caldara M Pochet N Beauvais A Guadagnini S Yan C Vinces MD Jansen A Prevost MC Latge J Fink GR Foster KR Verstrepen KJ FLO1 is a variable green beard gene that drives biofilm-like cooperation in budding yeast Cell (Cambridge, MA, U S) 2008 135 726 737 10.1016/j.cell.2008.09.037 PMC2703716 19013280

[B23] Stratford M Evidence for two mechanisms of flocculation in Saccharomyces cerevisiae Yeast 1989 5 S441 S445 2665372

[B24] Teixeira JA Mota M Goma G Continuous ethanol production by a flocculating strain of Kluyveromyces marxianus: bioreactor performance Bioprocess Eng 1990 5 123 127 10.1007/BF00388191

[B25] Van Mulders SE Christianen E Saerens SMG Daenen L Verbelen PJ Willaert R Verstrepen KJ Delvaux FR Phenotypic diversity of Flo protein family-mediated adhesion in Saccharomyces cerevisiae FEMS Yeast Res 2009 9 178 190 10.1111/j.1567-1364.2008.00462.x 19087208

[B26] Zhao XQ Bai FW Yeast flocculation: New story in fuel ethanol production Biotechnol Adv 2009 27 849 856 10.1016/j.biotechadv.2009.06.006 19577627

[B27] Zink RT Chatterjee AK Cloning and expression in Escherichia coli of pectinase genes of Erwinia carotovora subsp. carotovora Appl Environ Microbiol 1985 49 714 717 3888112 10.1128/aem.49.3.714-717.1985 PMC373578

